# Role of the UPS in Liddle syndrome

**DOI:** 10.1186/1471-2091-9-S1-S5

**Published:** 2008-10-21

**Authors:** Daniela Rotin

**Affiliations:** 1 Program in Cell Biology, The Hospital for Sick Children, and Biochemistry Department, University of Toronto, Ontario, M5G 1X8, Canada

## Abstract

Hypertension is a serious medical problem affecting a large population worldwide. Liddle syndrome is a hereditary form of early onset hypertension caused by mutations in the epithelial Na^+ ^channel (ENaC). The mutated region, called the PY (Pro-Pro-x-Tyr) motif, serves as a binding site for Nedd4-2, an E3 ubiquitin ligase from the HECT family. Nedd4-2 binds the ENaC PY motif via its WW domains, normally leading to ENaC ubiquitylation and endocytosis, reducing the number of active channels at the plasma membrane. In Liddle syndrome, this endocytosis is impaired due to the inability of the mutated PY motif in ENaC to properly bind Nedd4-2. This leads to accumulation of active channels at the cell surface and increased Na^+ ^(and fluid) absorption in the distal nephron, resulting in elevated blood volume and blood pressure. Small molecules/compounds that destabilize cell surface ENaC, or enhance Nedd4-2 activity in the kidney, could potentially serve to alleviate hypertension.

Republished from Current BioData's Targeted Proteins database (TPdb; ).

## Protein pathway involvement in disease

### Introduction

Elevated arterial blood pressure, or hypertension, poses a serious public health problem, affecting approximately 25% of the adult population in the industrial world [1], and becoming, along with obesity, a serious health problem in the developing world as well. In recent years, the causes of several genetic disorders leading to hypertension or hypotension have been identified, and deleterious mutations have been mapped to components of the aldosterone pathway, as well as to key ion channels and transporters expressed along the nephron. Prominent examples include Bartter syndrome type I, II or III, Gitelman syndrome, pseudohypoaldosteronism I (PHAI) and Liddle syndrome [2]; the latter two are associated with mutations in the epithelial Na^+ ^channel (ENaC) and are discussed in this review, with a particular focus on Liddle syndrome, a hereditary form of hypertension.

### The epithelial Na^+ ^channel

The amiloride-sensitive ENaC is an ion channel expressed in Na^+^-transporting epithelia such as those present in the distal nephron, respiratory epithelium, distal colon and taste buds [3]. In the kidney, it is primarily expressed in the distal connecting tubules (CNTs) and cortical collecting tubules (CCTs) of the nephron [4,5], where it provides the rate limiting step for Na^+ ^(and fluid) reabsorption into the blood stream [3,6,7]. This regulation of Na^+ ^and fluid absorption is tightly controlled by the hormones aldosterone (i.e. the renin-angiotensin-aldosterone pathway) and vasopressin (antidiuretic hormone, ADH), which stimulate channel activity [6,8]. The single channel characteristics of ENaC reveal high selectivity for Na^+ ^over K^+^, low single channel conductance (~5 pS), high sensitivity to amiloride (~100 nM) and slow gating [6]. ENaC activity is primarily regulated by control of its opening (Po) and numbers at the plasma membrane [8].

ENaC is comprised of three subunits, α, β and γ [9], each consisting of two transmembrane domains flanked by a large extracellular loop and two intracellular N- and C-termini, and is preferentially assembled at a stoichiometry of α_2_βγ [10,11] (although other configurations have been proposed [12,13]). Maximal channel activity is obtained when all three subunits are expressed together, but expression of α alone, or a combination of αβ, or αγ, results in low or moderate channel activity, respectively [9].

Genetic disease-causing mutations in ENaC, as well as mouse models, have shed important light on ENaC function and the pathology of ENaC-related diseases. For example, loss of function mutations in either α, β, or γ ENaC cause PHAI [14,15], a salt-wasting disease leading to hypotension, which is also mimicked in knockout mouse models lacking β or γ ENaC [16,17], or models expressing reduced levels of α ENaC [18], all of which exhibit reduced levels of channel expression and activity [9,19]. In contrast to the ENaC loss of function mutations causing PHAI, gain of function mutations in this channel cause Liddle syndrome.

### Liddle syndrome

Liddle syndrome (pseudoaldosteronism, OMIM 177200) is an autosomal dominant disease leading to early onset of hypertension. It is associated with hypokalemic alkalosis, reduced plasma rennin activity and low plasma aldosterone levels [20]. Over the past 12 years, work from Lifton's group (Yale University) and others has identified several deletions/mutations that cause Liddle syndrome, all of which map to β or γ ENaC and lead to elevated channel numbers and activity at the plasma membrane, as assessed by heterologously expressing these mutant ENaCs in *Xenopus *oocytes or cultured mammalian cells [2,21,22]. These genetic defects either delete the C-terminus of β or γ ENaC [23,24], or mutate a proline or a tyrosine within a short sequence, called the PY (Pro-Pro-x-Tyr) motif [25-28]. The PY motif, or extended PY motif (PPxYxxL [21,29]), is highly conserved in the C-termini of all ENaC subunits [26], and serves as a binding site for the Nedd4 family of ubiquitin ligases [30], as assessed by *in vitro *binding, yeast two-hybrid and co-immunoprecipitation assays, as well as by structural analysis (e.g. [29,30]).

### Regulation of ENaC by the ubiquitin system and its impairment in Liddle syndrome

Ubiquitylation, carried out by the sequential activity of E1, E2 and E3 (ubiquitin ligase) enzymes, usually regulates stability of target proteins that are tagged with ubiquitin by the E3 ligases [31]. Most of these proteins are degraded by the proteasome [32]. Recent studies have demonstrated, however, that ubiquitylation of transmembrane proteins can tag them for endocytosis and/or vesicular sorting, often resulting in their degradation in the lysosome [33,34]. This is usually achieved by the presence of ubiquitin binding motifs or domains (e.g. UIM, UBA, CUE, GAT, UEV, VHS) within proteins such as epsin/Eps15, Hrs and GGA, which function to recognize the ubiquitylated transmembrane proteins and facilitate their endocytosis or sorting [35].

**Figure 1:**
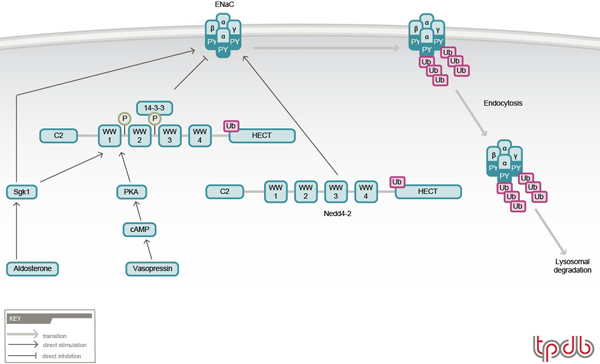
**Regulation of ENaC by Nedd4-2 in homeostasis**. The ubiquitin ligase Nedd4-2 binds (via its WW domains) to the PY motifs of ENaC, in turn ubiquitylating and targeting ENaC for endocytosis and lysosomal degradation. This process can be inhibited by Sgk1- or Akt-mediated phosphorylation of Nedd4-2, which leads to binding of 14-3-3 proteins to phosphorylated Nedd4-2, thus preventing Nedd4-2 from associating with ENaC and thus increasing ENaC levels at the plasma membrane. Sgk1 can also upregulate ENaC independently of Nedd4-2.

**Figure 2:**
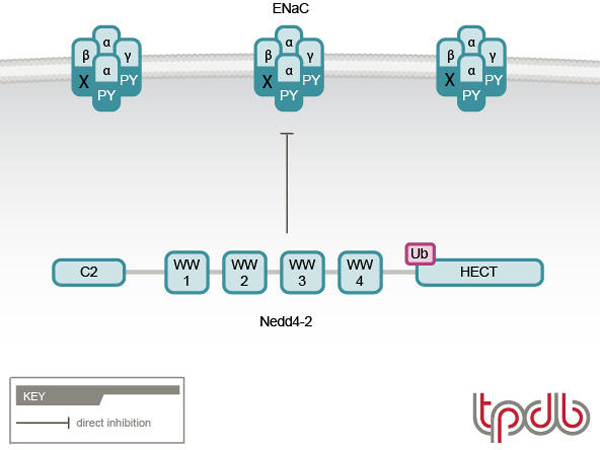
**Regulation of ENaC by Nedd4-2 and its impairment in Liddle syndrome**. In Liddle syndrome, deletion/mutation of the PY motif in βENaC (or γENaC, not shown) impairs the ability of Nedd4-2 to bind (and thus ubiquitylate) ENaC, leading to accumulation of ENaC channels at the plasma membrane and increased channel activity. (Modified with permission from Staub and Rotin).

Nedd4 family members are E3 ubiquitin ligases that comprise a C2 domain responsible for membrane targeting [36,37], three to four WW domains that bind the PY motifs of ENaC [29,30,38-42], and a ubiquitin ligase HECT (homologous to E6AP carboxyl-terminus) domain [43,44] (Figures 1 and 2). Of the two closely related Nedd4 members, Nedd4-1 and Nedd4-2, the latter binds ENaC more strongly due to the presence of an additional, high affinity WW domain (WW3, out of four WW domains) [41,42]. Accordingly, Nedd4-2 was shown to effectively suppress ENaC activity by enhancing removal of the channel from the plasma membrane [45-47], and ubiquitylation of ENaC was demonstrated to destabilize cell surface ENaC [48] (Figure 2). Indeed, our recent work has demonstrated that Nedd4-2 can ubiquitylate ENaC present at the apical membrane of cultured kidney epithelial cells [49]. The few Nedd4-1 proteins that also contain this high affinity WW3 domain (e.g. human and *Drosophila *Nedd4-1) are also able to suppress ENaC activity when heterologously expressed in *Xenopus *oocytes or cultured cells, although in some cases this is prevented in the presence of the C2 domain (for example, in the case of human Nedd4-1 [41,47,50]), possibly (albeit speculatively) due to inhibitory interactions between the C2 and HECT domains.

Experiments performed in *Xenopus *oocytes or mammalian cultured cells that ectopically express ENaC reveal that unlike the ability of Nedd4-2 to induce removal of wild-type ENaC from the plasma membrane by ubiquitylation (likely linked to subsequent clathrin-mediated endocytosis [51]), Liddle syndrome mutations in the PY motif of β or γ ENaC severely attenuate this removal, leading to increased retention of mutant ENaC at the cell surface and to elevated channel activity [45,46,49] (Figure 2). Accordingly, conditional knockout mice bearing a deletion of the PY motif in βENaC (a mouse model for Liddle syndrome) develop hypertension that is induced by high salt diet [52]. Moreover, channel feedback inhibition by elevated intracellular Na^+ ^concentrations exhibited by wild-type ENaC (demonstrated *ex vivo *in the CCTs of rats [53]) is defective in ENaC bearing the Liddle syndrome mutations in the PY motif (as shown by ectopic expression of PY motif-mutated ENaC in *Xenopus *oocytes [54]), further exacerbating Na^+ ^loading. Together, this results in increased Na^+ ^and fluid reabsorption in the distal nephron, and increased blood volume and blood pressure, which are hallmarks of Liddle syndrome.

As indicated in the section *The epithelial Na*^+^*channel*, ENaC is tightly (positively) regulated in the kidney by the mineralocorticoid hormone aldosterone. One of the recently discovered aldosterone targets is Sgk1, a Ser/Thr kinase from the Akt (PKB) family that was found to elevate ENaC levels/activity in response to aldosterone in rat or mouse kidney and in A6 cells (a *Xenopus *cell line endogenously expressing ENaC and responsive to aldosterone) [55,56]. This effect can be mediated either without (see below) or via regulation of Nedd4-2 (Figure 1). Nedd4-2 (but not Nedd4-1) possesses Sgk1 phosphorylation sites and, when phosphorylated by Sgk1 (in cultured cells), is prevented from downregulating ENaC, leading to increased ENaC retention at the cell surface and thus increased ENaC activity [57,58]. This effect is believed to be mediated by association of the adaptor protein 14-3-3, known to bind phosphoSer/Thr [59], with Ser-phosphorylated Nedd4-2, thus preventing Nedd4-2 from binding ENaC [60,61] (by as yet unknown mechanism(s)). In support, the expression of 14-3-3β and Nedd4-2, as well as Nedd4-2 phosphorylation, were recently shown to be induced in CCT cells by dietary salt and by aldosterone [62-64]. However, aldosterone and Sgk1 can stimulate ENaC independently of Nedd4-2 and their role in Liddle syndrome is controversial: aldosterone and Sgk1 were found to increase cell surface abundance of ENaC channels bearing Liddle syndrome deletions/mutations (which cannot bind Nedd4-2) [65-68] and, importantly, CCTs harvested from mutant mice bearing a Liddle syndrome deletion [52] revealed a normal response to aldosterone [69].

In addition to aldosterone, the hormone vasopressin also increases ENaC activity (as well as water absorption) in the distal nephron by binding to V_2 _receptors and stimulating activation of adenylate cyclase and the release of cAMP [6]. cAMP increases the density of ENaC channels (endogenously or ectopically expressed in epithelial cells) at the plasma membrane [70,71], an effect suggested to be impaired in channels bearing the Liddle syndrome PY motif mutations due to defective trafficking to the cell surface [72], or mobilization from a sub-apical pool [49]. Recent studies also suggested that Nedd4-2 phosphorylation by PKA (which is activated by cAMP) provides inhibitory function much like Sgk1, thus inhibiting the ability of Nedd4-2 to suppress ENaC, leading to increased cell surface abundance of this channel [73].

Among the other factors that regulate ENaC (aside from hormones, ions and Nedd4-2), are proteases such as CAP proteins and TMPRSS3, which activate ENaC by proteolytic cleavage of its ectodomains [3,74-77]. A recent paper suggests that Liddle syndrome mutations increase the number of cleaved (active) ENaCs at the cell surface (thus further increasing Na^+ ^absorption), and that Nedd4-2 and ENaC ubiquitylation regulate the number of cleaved channels at the plasma membrane [78].

## Disease models, knockouts and assays

To date, only one mouse model for Liddle syndrome has been generated. These mice, created by the Rossier/Hummler groups (Institut de Pharmacologie et de Toxicologie, Switzerland) in 1999, bear a deletion of the PY motif in βENaC (a stop codon is inserted at a residue corresponding to residue Arg566 in human βENaC, as found in the original pedigree described by R. Lifton [23]). The mice have no phenotype under a normal salt diet, but develop hypertension when fed a high salt diet [52]. To date, no other relevant knockout mice of Liddle syndrome have been developed.

## Disease targets and ligands

Liddle syndrome patients are treated with the ENaC antagonist amiloride-triamterene and a low salt diet to stabilize their high blood pressure. While Liddle syndrome is a rare disorder, as are several genetic forms of hypertension [2], other forms of hypertension are very common in the population and have no known genetic components. Inhibiting ENaC activity, the rate limiting step in the regulation of Na^+ ^and fluid reabsorption in the nephron, could provide an attractive target to treat hypertension. With the advent of high throughput technology it is possible to test for inhibition of ENaC activity by screening with small molecules/compound libraries, with the hope of identifying inhibitory compounds that may be superior to amiloride and its analogs. In that regard, we have recently developed a high throughput assay that allows quantification of the amounts of cell surface ENaC (Chen and Rotin, unpublished). Given the key role played by the ubiquitin system/Nedd4-2 in regulating ENaC cell surface stability and ENaC function, identifying compounds that destabilize/decrease ENaC levels at the plasma membrane could have potential therapeutic benefits for the treatment of hypertension. Stimulating Nedd4-2 activity, which leads to ENaC endocytosis/degradation, could also be a possibility. However, since Nedd4-2 likely has other targets in other tissues/cells, this approach needs to be scrutinized to ensure it is targeted specifically to ENaC in the kidney. It is likely that use of putative compounds that aim to enhance ENaC internalization or Nedd4-2 activity would be more effective towards other forms of hypertension and not Liddle syndrome, since the latter carries mutations that already inhibit ENaC internalization and are insensitive to Nedd4-2.

## New frontiers in drug discovery

Despite significant recent advances, several key questions remain to be answered regarding the regulation of ENaC by Nedd4-2 and the ubiquitin system. These include:

(i) How is sensing of elevation of intracellular concentrations of Na^+ ^(that normally shuts down ENaC) related to the Liddle syndrome mutations? If this is regulated via Nedd4-2, how is Nedd4-2 (directly or indirectly) able to sense Na^+^?

(ii) How does phosphorylation of Nedd4-2 by Sgk1 (on sites not within the WW domains), which leads to binding of 14-3-3 to Nedd4-2, inhibit the association of Nedd4-2 with ENaC, which is mediated via the WW domains?

(iii) What is the exact stoichiometry of Nedd4-2-ENaC interactions, and how is it that loss of only one PY motif is sufficient to cause Liddle syndrome?

(iv) How is the activity of Nedd4-2 itself regulated in the cell?

(v) What is the physiological function of Nedd4 proteins *in vivo *in mammals, especially in the kidney? The latter should be answered with the generation of knockout murine models for these proteins (not yet published). Future work will undoubtedly address these and other important questions that investigate the relationship between ENaC and the ubiquitin system.

## List of abbreviations used

CCT: cortical collecting tubule; CNT: distal connecting tubule; ENaC: epithelial Na^+ ^channel; HECT: homologous to E6AP carboxyl-terminus; PHAI: pseudohypoaldosteronism I; PY: Pro-Pro-x-Tyr.

## Competing interests

The author declares that they have no competing interests.

## Publication history

Republished from Current BioData's Targeted Proteins database (TPdb; ).
